# Double Jeopardy: A Distinct Mortality Pattern Among Preterm Infants with Congenital Heart Disease

**DOI:** 10.1007/s00246-024-03519-4

**Published:** 2024-06-12

**Authors:** Brennan V. Higgins, Philip T. Levy, Molly K. Ball, Minso Kim, Shabnam Peyvandi, Martina A. Steurer

**Affiliations:** 1 Department of Pediatrics, University of California San Francisco, 550 16th Street, 5th Floor, San Francisco, CA 94143 USA; 2 Department of Pediatrics, Boston Children’s Hospital and Harvard Medical School, Boston, MA USA; 3 Department of Pediatrics, The Ohio State University College of Medicine, Columbus, OH USA; 4 Department of Epidemiology and Biostatistics, California Preterm Birth Initiative, University of California San Francisco, San Francisco, CA USA

**Keywords:** Congenital heart disease, Preterm birth, Mortality, Survival

## Abstract

Contemporary United States (US) data on the survival of preterm infants with congenital heart disease (CHD) are unavailable despite the over-representation of CHD and improving surgical outcomes in the preterm population. The aim of this study is to use population-based data to compare 1-year survival and early mortality (< 3 days) by gestational age (GA) between preterm infants with and without cyanotic CHD (CCHD) in the US. This national retrospective cohort included all liveborn, preterm infants between 21 and 36 weeks GA with a birth certificate indicating the presence or absence of CCHD (*n* = 2,654,253) born between 2014 and 2019 in the US. Data were provided by the US Center for Disease Control database linking birth and death certificates. Of liveborn preterm infants, 0.13% (*n* = 3619) had CCHD. 1-year survival was significantly lower in infants 23–36 weeks with CCHD compared to those without. The greatest survival gap occurred between 28 and 31 weeks (28 weeks adjusted risk difference 37.5%; 95% CI 28.4, 46.5; 31 weeks 37.9%; 30.5, 45.3). Early mortality accounted for more than half of deaths among infants 23–31 weeks with CCHD (23 weeks-68%, CI 46.7, 83.7; 31 weeks-63.9%, 52.9, 73.6). Survival trends demonstrated worsened 1-year survival in infants 35–36 weeks with CCHD over the study period. The pattern of mortality for preterm infants with CCHD is distinct from those without. The significant survival gap in the very preterm population and notably high rate of early death in the infants with CCHD calls for renewed attention to early neonatal intensive care for this dually affected population.

**Supplementary Information:**

The online version contains supplementary material available at 10.1007/s00246-024-03519-4.

## Introduction

Congenital malformations and prematurity are the two leading causes of infant mortality in the United States (US) (21% and 16%, respectively) [1]. Congenital heart disease (CHD) is the most common type of congenital malformation affecting approximately 1% of births annually in the US [2]. CHD is overrepresented in preterm compared to term infants with prevalence estimates increasing as much as five-fold with decreasing gestational age (GA) [3, 4].

Preterm infants with CHD experience double jeopardy—the mortality and morbidity of prematurity that is inversely related to GA [5, 6] as well as that of CHD. The excess mortality experienced by preterm infants with CHD varies by cardiac defect [3] and is inversely related to GA [4]. Nevertheless, there is a paucity of literature looking at mortality using GA as a continuous variable rather than dichotomizing term and preterm mortality [4, 7]. Additionally, data about extremely preterm infants < 28 weeks with CHD are rare [4, 7].

Although post-surgical mortality has been reported as higher in preterm compared to term infants, with one study estimating a six-fold increase in mortality risk for preterm infants in the 6 months following cardiac surgery [8], early mortality is less well understood. Early neonatal mortality, defined as less than 1 week, was noted to be significant in one international study [9]. However, contemporary US national trends are unknown.

This study therefore aimed to use contemporary US national population-based data to (1) describe differences in 1-year survival between preterm infants with and without cyanotic congenital heart disease (CCHD) by week of gestation, (2) understand the extent of early mortality (< 3 days), and (3) compare mortality trends between preterm neonates with and without CCHD from 2014 to 2019.

## Methods

The Center for Disease Control and Prevention (CDC) provides yearly cohort linked birth/infant death public use files [10]. These datasets contain all live births in the US and are linked to death certificates during the first year of life. In 2014, the universal adoption of the revised birth certificate (introduced initially in 2003) became mandatory in all states. This included documentation of the presence or absence of CCHD. Thus, this study includes birth data files beginning in January 2014 and ending in December 2019, the last publicly available data at the time of the analysis.

All liveborn, preterm infants (i.e., born < 37 weeks of gestation) are included in this population-based, retrospective cohort analysis. Infants with GA at birth < 21 weeks or for whom CCHD status was unknown or not reported on the birth certificate were excluded from this study.

### Predictors

The predictor variable is the presence or absence of CCHD as documented on the birth certificate. There is no further information available from this database about the specific type of CHD, nor is there any data for non-cyanotic CHD.

Per the data dictionary [10], the GA variable is a combination of the clinical estimate and the best obstetric estimate. For time trend analyses, GA at birth was categorized into the following groups: ≤ 25 weeks, 25–28 weeks, 29–31 weeks, 32–34 weeks, and 35–36 weeks.

Z-score for birth weight was calculated based on sex and GA using the Fenton method [11]. Intrauterine growth restriction (IUGR) was defined, based on convention, as a weight less than the 10th percentile for gestational age corresponding to a z-score less than negative 1.3.

### Outcomes

The primary outcome was 1-year survival. The secondary outcome was early mortality defined as mortality at less than three days of age. Three days of age was chosen to avoid confounding early mortality with operative mortality, since only a very small percentage of CCHD patients undergo urgent surgery within the first 72 h after birth, especially when born at preterm GAs.

### Statistical analysis

Baseline characteristics between infants with and without CCHD were compared using a t test for continuous variables and chi-square test for categorical variables.

We present proportion of infants who survived to 1 year of age and proportion who died before three days of age (early mortality) with 95% confidence interval (CI) by GA at birth for neonates with and without CCHD. We calculated crude and adjusted risk differences to compare survival and mortality between infants with and without CCHD by GA. Analyses were adjusted for sex, multiple gestation, and IUGR. We graphed adjusted risk differences by GA for infants with and without CCHD. All the confounders were kept at their mean value.

To assess change in mortality over time, we calculated crude and adjusted risk differences per year separately for neonates with and without CCHD by GA categories. For this analysis, we fit logistic models with mortality as the outcome and year of birth as our primary predictor. We used year as a linear variable and adjusted for sex, multiple gestation, and IUGR.

A *P* value of < 0.05 was considered significant for all analyses. All analyses were performed using Stata version 16.1 (Stata Statistical Software: Release 16. College Station, TX: StataCorp LP). As the study utilized a publicly available dataset, it was exempt from institutional reviewing board review.

## Results

**Fig. 1:**
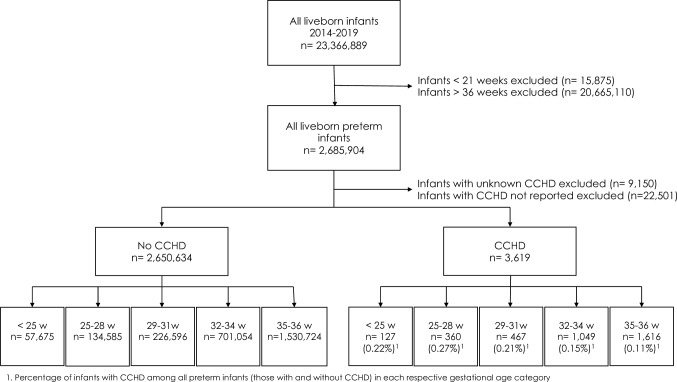
Study population

During the study period 2,685,904 preterm infants 21–36 weeks GA were born in the US (11.5% of all live births). Of those, 2,654,253 had an indication of the presence or absence of CCHD on their birth certificate and were included in this study. A total of 0.13% (*n* = 3619) of all liveborn preterm infants had documented CCHD. The prevalence of CCHD was highest among 25–28-week infants (0.27%) and lowest in 35–36-week infants (0.11%) (Fig. 1).

Preterm infants with CCHD were less likely to be multiple gestation (14.8% versus 17.2%, *p* < 0.001), more likely to be IUGR (20.7% versus 8.5%, *p* < 0.001), and were born at a lower GA (median 34 weeks, IQR 31–36 weeks versus median 35 weeks, IQR 33–36 weeks, *p* < 0.001) compared to infants without CCHD (Table 1). Health conditions including pre-existing diabetes, gestational diabetes, pre-existing hypertension, and gestational hypertension were more prevalent in individuals with newborns with CCHD (Table 1). Individuals with newborns with CCHD were also more likely to have private insurance and to self-identify as Non-Hispanic White (Table 1).

**Table 1:** Baseline characteristics of the study population

		No CCHD	CCHD	*p*-value
		*n* = 2,650,634	*n* = 3619	
Maternal race/ethnicity	NHW	1,222,025 (46.1%)	2043 (56.5%)	< 0.001
	NHB	552,255 (20.8%)	528 (14.6%)	
	NH AIAN	26,011 (1.0%)	49 (1.4%)	
	NH Asian	140,379 (5.3%)	159 (4.4%)	
	NHOPI	8958 (0.3%)	17 (0.5%)	
	NH other	57,611 (2.2%)	90 (2.5%)	
	Hispanic	618,974 (23.4%)	659 (18.2%)	
	Unknown	24,421 (0.9%)	74 (2.0%)	
Maternal education	did not complete High school	436,029 (16.7%)	547 (15.5%)	0.20
	Completed high school	1,496,512 (57.3%)	2035 (57.6%)	
	Bachelor or Master’s degree	621,660 (23.8%)	868 (24.6%)	
	Doctoral degree	57,128 (2.2%)	84 (2.4%)	
Insurance status	Private Insurance	1,357,724 (51.2%)	1997 (55.2%)	< 0.001
	Medicaid	1,292,910 (48.8%)	1622 (44.8%)	
Pre-existing Diabetes	Present	53,280 (2.0%)	237 (6.5%)	< 0.001
	Absent	2,594,703 (97.9%)	3375 (93.3%)	
	Unknown	2651 (0.1%)	7 (0.2%)	
Gestational Diabetes	Present	211,689 (8.0%)	337 (9.3%)	0.003
	Absent	2,436,294 (91.9%)	3275 (90.5%)	
	Unknown	2651 (0.1%)	7 (0.2%)	
Pre-existing Hypertension	Present	106,138 (4.0%)	216 (6.0%)	< 0.001
	Absent	2,541,845 (95.9%)	3396 (93.8%)	
	Unknown	2651 (0.1%)	7 (0.2%)	
Gestational Hypertension	Present	351,267 (13.3%)	534 (14.8%)	0.006
	Absent	2,296,716 (86.6%)	3078 (85.1%)	
	Unknown	2651 (0.1%)	7 (0.2%)	
Maternal BMI, median (IQR)		26.3 (22.3, 32.1)	26.4 (22.2, 32.4)	0.79
Maternal BMI > 35	Present	465,410 (17.6%)	660 (18.2%)	0.28
	Absent	2,185,224 (82.4%)	2959 (81.8%)	
Gestation	Singleton	2,196,040 (82.8%)	3084 (85.2%)	< 0.001
	Multiple birth	454,594 (17.2%)	535 (14.8%)	
Sex	Male	1,408,070 (53.1%)	1978 (54.7%)	0.065
	Female	1,242,564 (46.9%)	1641 (45.3%)	
Gestational age (weeks), median (IQR)		35.0 (33.0, 36.0)	34.0 (31.0, 36.0)	< 0.001
Z-score for BW, median (IQR)		0.2 (− 0.5, 1.2)	− 0.2 (− 1.1, 0.8)	< 0.001
IUGR	Absent	2,424,529 (91.5%)	2870 (79.3%)	< 0.001
	Present	226,105 (8.5%)	749 (20.7%)	

*CCHD* cyanotic congenital heart disease, *NHW* Non-Hispanic White, *NHB* Non-Hispanic Black, *NH AIAN* Non-Hispanic American Indian or Alaska Native, *NH Asian* Non-Hispanic Asian, *NHOPI* Native Hawaiian and Other Pacific Islanders, *NH Other* Non-Hispanic Other, *IQR* interquartile range, *BW* birth weight, *IUGR* intrauterine growth restriction, *BMI* Body Mass Index

### 1-Year Survival

**Fig. 2:**
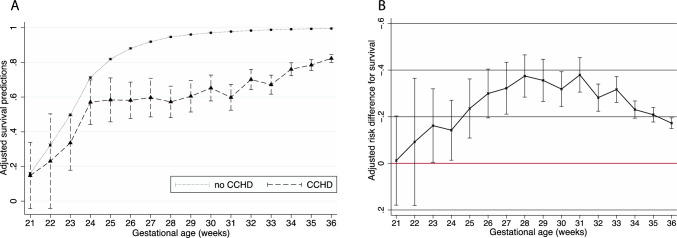
Adjusted predictions and absolute adjusted risk difference for 1-year survival in infants with and without CCHD by gestational age

There were distinct trends in 1-year survival by GA for infants with versus without CCHD. In neonates without CCHD, 1-year survival increased rapidly from 18.1% (CI 17.3, 18.9) at 21 weeks to 79.3% (CI 78.8, 79.8) at 25 weeks and was > 90% after 27 weeks (Fig. 2a, Supplemental Table 1). Among infants with CCHD, one-year survival improved with GA from 21 weeks (16.7%, CI 3.5, 52.4) to 24 weeks (53.2%, CI 19.1, 49.5), plateaued from 24 to 31 weeks (54.4%, CI 47.1, 61.5), and improved again thereafter through 36 weeks (76.8%, CI 74.0, 79.3) (Fig. 2a, Supplemental Table 1).

The adjusted risk difference for 1-year survival between infants with and without CCHD was statistically significant for all GAs between 23 and 36 weeks (Fig. 2b, Supplemental Table 1). The magnitude of the adjusted risk difference was greatest from 28 weeks (37.5%, CI 28.4, 46.5) to 31 weeks (37.9%, CI 30.5, 45.3) (Fig. 2b, Supplemental Table 1).

### Early Mortality < 3 Days of Age

**Fig. 3:**
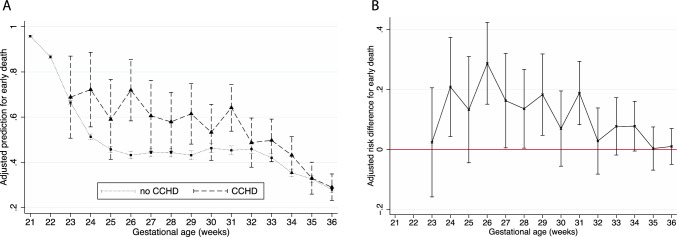
Adjusted prediction and absolute adjusted risk difference for early mortality in infants with and without CCHD by gestational age

Among infants who died, the proportion who died before three days of age rapidly declined from > 90% with advancing GA for preterm infants without CCHD, reaching 46.4% (CI 45.0, 47.7) by 25 weeks (Fig. 3a, Supplemental Table 2). All infants born at 21 and 22 weeks with CCHD who died did so before three days of age. Early mortality in infants with CCHD accounted for more than half and up to nearly three quarters of deaths between 23 weeks (68%, CI 46.7, 83.7) and 31 weeks GA (63.9%, CI 52.9, 73.6) and only declined thereafter (Fig. 3a, Supplemental Table 2). The adjusted early mortality risk difference between infants with and without CCHD was largest at 24 weeks (20.8%, CI 4.3, 37.4) and 26 weeks (28.7%, CI 15.1, 42.4) and was no longer statistically significant by 32 weeks (Fig. 3b, Supplemental Table 2).

### Time Trends

Over the study period, a small but statistically significant annual decrease was observed in the adjusted 1-year mortality risk for infants without CCHD in most GA categories. It was largest in the neonates born at < 25 weeks GA (− 0.9% decrease in mortality per year, CI − 1.1, − 0.6) (Table 2). In contrast, among neonates with CCHD, there was a trend toward increased 1-year mortality in all GA categories over time. However, this only reached statistical significance in GA 35–36 weeks (1.3% increase in mortality per year; CI 0.08, 2.5) (Table 2).

**Table 2:** Infant mortality/survival trends over time for preterm neonates with and without CCHD by gestational age groups

	Neonates with CCHD		Neonates without CCHD	
	Crude risk difference	Adjusted* risk difference	Crude risk difference	Adjusted* risk difference
Gestational age				
≤ 25 weeks	1.4 (− 3.7, 6.5)	1.2 (− 4.1, 6.5)	− ***0.9 (***− ***1.1, ***− ***0.6)***	− ***0.9 (***− ***1.1, ***− ***0.6)***
25–28 weeks	2.5 (− 0.5, 5.5)	2.1 (− 1.0, 5.2)	− ***0.3 (***− ***0.4, ***− ***0.2)***	− ***0.3 (***− ***0.4, ***− ***0.2)***
29–31 weeks	***3.0 (0.4, 5.7)***	2.7 (− 0.05, 5.4)	− 0.01 (− 0.05, 0.02)	− 0.009 (− 0.004, 0.003)
32–34 weeks	− 0.2 ( − 1.8, 1.4)	− 0.4 (− 2.0, 1.3)	− 0.001 (− 0.993, 0.0004)	− ***0.002 (***− ***0.003, ***− ***0.001)***
35–36 weeks	***1.3 (0.1, 2.5)***	***1.3 (0.08, 2.5)***	− ***0.008 (***− ***0.002, ***− ***0.0008)***	− ***0.007 (***− ***0.01, ***− ***0.001)***

*Adjusted for sex, multiple gestation, and IUGR (z-score for BW < − 1.3)

Risk difference is given per year (2014–2019, total of 5), a positive number indicates a risk increase in mortality (i.e., a decrease in survival chance), and a negative number indicates a risk decrease in mortality (i.e., increase in survival chance)

Bold and italic results are statistically significant with *p*-value < 0.05

## Discussion

This national retrospective cohort study is the first to provide contemporary population-based US demographic and survival statistics for preterm infants with CCHD by week of gestation at birth, including extremely preterm infants. This study suggests that the pattern of mortality for preterm infants with CCHD is distinct from that of preterm infants without CCHD. Understanding these patterns in this understudied group of vulnerable infants with national, population-based data are extremely valuable despite inherent limitations in birth certificate data and is critical to advancing care.

CCHD was over represented in preterm infants, with the highest prevalence occurring in infants born between 25 and 28 weeks GA. Preterm infants with CCHD born ≥ 23 weeks GA were less likely to survive to 1 year compared to GA-matched infants without CCHD. The largest survival gap occurred between 28 and 31 weeks GA. Between 23- and 31-week GA, early mortality accounted for more than half of the deaths in infants born with CCHD, substantially more than those without CCHD. Finally, 1-year survival of preterm infants born with CCHD in the US did not improve over the study period and in fact worsened in 35–36-week infants. In contrast, survival improved in almost all GA categories among preterm infants without CCHD.

Infants with CCHD in this study were more likely to be born premature, IUGR, and to birthing persons with pre-existing or antepartum health conditions. It is known that infants with CHD are more likely to be preterm [3, 4] and growth restricted [12]. Moreover, these factors are predictive of poorer outcomes [3, 4, 13]. Additionally, health complications including hypertension and diabetes are known to be more common in pregnancies with CHD [14, 15], but their impact on outcomes in infants with CHD is less clear [16].

### 1-Year Survival

It is well established that preterm infants with CHD are less likely to survive than preterm infants without CHD [17]. However, the majority of studies use GA as a dichotomous variable (term/preterm), and few studies have evaluated survival by GA as a continuous variable and with the inclusion of extremely preterm infants. With this unique lens, this study was able to demonstrate that differences in survival between preterm infants with and without CCHD were not consistent across gestational ages. At the extreme end of viability (< 23 weeks), the difference between the two groups was the smallest. Given the known high mortality rates in this periviable population [5]_,_ it is likely that mortality in these infants was primarily driven by extreme prematurity. The survival gap increased from 24 to 28 weeks, was widest from 28 to 31 weeks, and decreased modestly, but remained present thereafter. The widening gap in 1-year survival from 24 to 31 weeks GA is driven by an asymptotic improvement in survival in infants not affected by CCHD with a relative stagnation of the probability of survival in infants with CCHD.

An international cohort study of infants 24–31 weeks GA with severe CHD also found no significant difference in mortality between infants with and without CHD at the youngest GAs studied, 24–26 weeks, but an increasing survival gap thereafter [7]. Similarly, a California cohort study published by our group demonstrated an increasing survival gap between infants with and without serious CHD in infants ≥ 26 weeks GA up until 31 weeks GA and a slight decrease thereafter [4]. This California cohort study, however, did not include infants < 26 weeks. Notably, point estimates for mortality are lower in these two studies [4, 7] compared to our study likely due to inclusion of non-cyanotic CHD. Other recent studies combine GAs < 32 weeks [9, 18] making it difficult to ascertain these GA-specific trends.

There is much to gain to mirror the improved outcomes of infants born at preterm GAs without CCHD. The field of neonatology has made progress in survival of preterm infants over the last few decades [5, 6, 19]. Even over the 5-year period from 2014 to 2019, 1-year survival improved in almost all GA categories among preterm infants without CCHD in this study. The same was not true for infants with CCHD. 1-year survival of preterm neonates with CCHD in the US did not improve over the study period and in fact, it worsened in 35- to 36-week infants. This lack of improvement on a national level is concerning. It may be due to the limits of surgical techniques but it may also be due to limitations of medical intensive care in the pre- or post-operative period and beyond.

### Early Mortality < 3 Days of Age

Neonates who die shortly after birth in the neonatal intensive care unit without ever being transferred to a cardiac intensive care unit or undergoing a surgical intervention are missed in existing CHD registry data. This represents a significant percentage of infants. In fact, early mortality, before three days of age, accounted for more than half of the deaths among infants with CCHD born at ≤ 31 weeks GA in this study. Similarly, the EPICARD population-based cohort from France looked at early neonatal mortality, defined as death less than 1 week of age, and also found that it accounted for more than half of neonatal deaths [9]. Therefore, one of the major benefits of using this population-based dataset is that it includes all liveborn preterm infants with CCHD.

The burden of early mortality among preterm infants with CCHD is striking, particularly among very preterm neonates. It may in part reflect decisions around the provision of active care. However, it also further underscores the need for improved early, pre-operative neonatal intensive care for this vulnerable population, many of whom can be stabilized on prostaglandin E — especially, as surgical techniques improve and more preterm infants are able to undergo technically successful cardiac surgery [8, 20].

One approach might be specialized units designed to care for this dually affected population where the complicated and interlacing physiology of congenital heart disease and comorbidities of prematurity, including fetal lung immaturity and neurologic vulnerability, can be considered and balanced by a skilled neonatal cardiac intensive care team. Small baby units have been successful in delivering evidence-based care and improving outcomes for extremely preterm infants [21, 22] and as such, represent an interesting model to consider for the preterm CHD population. A recent publication demonstrated success with this type of care model in reducing in-hospital mortality for very preterm infants with CHD at a single center [23].

### Strengths and Limitations

The main strengths of this study come from the nationally representative birth certificate data inclusive of all liveborn preterm infants with and without CCHD down to 21 weeks GA and available timing of death in days. This allows for a granular understanding of outcome metrics for this understudied group of vulnerable infants at a population level that is not well captured in cardiac registry data. Nevertheless, population-based data also comes with limitations. One of the main limitations of this study is the exclusion of all non-cyanotic CHD. Thus, the results of this study, particularly the survival predictions, are not generalizable to all CHD, and especially not to the milder diagnoses. Additionally, specific type of CCHD, major neonatal, or cardiac morbidities and actual timing of surgery are not available from the database. It is therefore impossible to actually distinguish between pre-operative mortality, operative mortality, and post-operative mortality. Furthermore, information around decisions related to providing or with-holding interventions including neonatal resuscitation is not available. A portion of infants in the early mortality group likely did not receive active care but the exact percentage cannot be known. Additionally, prenatal data are not available and thus fetuses with CHD who are not liveborn are excluded. Although rare, infants not identified to have cyanotic congenital heart disease during their initial admission, when birth certificates are completed, are also missed by this database. Finally, morbidity and longer-term neurologic outcomes are outside the scope of this work. They are, however, critically important considerations and have been previously reported to be poorer in infants with CHD [4, 24].

## Conclusion

The significant 1-year survival gap between preterm infants with and without CCHD, especially those born between 28 and 31 weeks GA, and the comparative lack of improvement in 1-year survival over time for infants with CCHD in the US highlights the need for improved intensive care for and targeted study of this vulnerable, dually affected population. The notably high rate of early mortality in preterm infants with CCHD born ≤ 31 weeks GA suggests improved immediate neonatal care in the transitional and pre-operative periods is imperative. Dedicated multidisciplinary neonatal cardiac intensive care units could be helpful in achieving this goal and lead to advancements in this emerging field.

## Supplementary Information

Below is the link to the electronic supplementary material.

Supplementary file1 (DOCX 19 KB)

## Abbreviations

CCHD: Cyanotic congenital heart disease
CDC: Center for disease control and prevention
CHD: Congenital heart disease
CI: Confidence interval
GA: Gestational age
IUGR: Intrauterine growth restriction
US: United States

## References

[1] Ely DM, Driscoll AK (2022) Infant mortality in the United States, 2020: data from the period linked birth/infant death file. Natl Vital Stat Rep 71(5):1–18 36190428

[2] Centers for Disease Control and Prevention. Data and Statistics on Congenital Heart Defects. https://www.cdc.gov/ncbddd/heartdefects/data.html#print. Accessed 16 April, 2024.

[3] Chu PY, Li JS, Kosinski AS, Hornik CP, Hill KD (2017) Congenital heart disease in premature infants 25–32 Weeks’ gestational age. J Pediatr 181:37-41.e1 27816222 10.1016/j.jpeds.2016.10.033 PMC5274591

[4] Steurer MA, Baer RJ, Chambers CD, Costello J, Franck LS, McKenzie-Sampson S et al (2021) Mortality and major neonatal morbidity in preterm infants with serious congenital heart disease. J Pediatr 239:110-116.e3 34454949 10.1016/j.jpeds.2021.08.039 PMC10866139

[5] Bell EF, Hintz SR, Hansen NI, Bann CM, Wyckoff MH, DeMauro SB et al (2022) Mortality, in-hospital morbidity, care practices, and 2-year outcomes for extremely preterm infants in the US, 2013–2018. JAMA 327(3):248–263 35040888 10.1001/jama.2021.23580 PMC8767441

[6] Patel RM (2016) Short- and long-term outcomes for extremely preterm infants. Am J Perinatol 33(3):318–328 26799967 10.1055/s-0035-1571202 PMC4760862

[7] Norman M, Håkansson S, Kusuda S, Vento M, Lehtonen L, Reichman B, Darlow BA, Adams M, Bassler D, Isayama T, Rusconi F, Lee S, Lui K, Yang J, Shah PS, International Network for Evaluation of Outcomes in Neonates (iNeo) Investigators (2020) Neonatal outcomes in very preterm infants with severe congenital heart defects: an international cohort study. J Am Heart Assoc 9(5):e015369 32079479 10.1161/JAHA.119.015369 PMC7335543

[8] Alarcon Manchego P, Cheung M, Zannino D, Nunn R, D’Udekem Y, Brizard C (2018) Audit of cardiac surgery outcomes for low birth weight and premature infants. Semin Thorac Cardiovasc Surg 30(1):71–78 29432888 10.1053/j.semtcvs.2018.02.013

[9] Laas E, Lelong N, Ancel PY, Bonnet D, Houyel L, Magny JF, Andrieu T, Goffinet F, Khoshnood B, EPICARD study group (2017) Impact of preterm birth on infant mortality for newborns with congenital heart defects: the EPICARD population-based cohort study. BMC Pediatr 17(1):124 28506266 10.1186/s12887-017-0875-z PMC5433049

[10] Vital Statistics Online Data Portal. https://www.cdc.gov/nchs/data_access/vitalstatsonline.htm. Accessed August 23, 2023.

[11] Fenton TR, Sauve RS (2007) Using the LMS method to calculate z-scores for the Fenton preterm infant growth chart. Eur J Clin Nutr 61:1380–1385 17299469 10.1038/sj.ejcn.1602667

[12] Ghanchi A, Rahshenas M, Bonnet D, Derridj N, LeLong N, Salomon LJ, Goffinet F, Khoshnood B (2021) Prevalence of growth restriction at birth for newborns with congenital heart defects: a population-based prospective cohort study EPICARD. Front Pediatr 28(9):676994 10.3389/fped.2021.676994 PMC8192794 34123973

[13] Steurer MA, Peyvandi S, Costello JM, Moon-Grady AJ, Habib RH, Hill KD, Jacobs ML, Jelliffe-Pawlowski LL, Keller RL, Pasquali SK, Reddy VM, Tabbutt S, Rajagopal S (2021) Association between Z-score for birth weight and postoperative outcomes in neonates and infants with congenital heart disease. J Thorac Cardiovasc Surg 162(6):1838–1847 33640137 10.1016/j.jtcvs.2021.01.065

[14] Helle E, Priest JR (2020) Maternal obesity and diabetes mellitus as risk factors for congenital heart disease in the offspring. J Am Heart Assoc 9(8):e011541 32308111 10.1161/JAHA.119.011541 PMC7428516

[15] Auger N, Fraser WD, Healy-Profitós J, Arbour L (2015) Association between preeclampsia and congenital heart defects. JAMA 314(15):1588–1598 26501535 10.1001/jama.2015.12505

[16] Steurer MA, Peyvandi S, Baer RJ, Oltman SP, Chambers CD, Norton ME, Ryckman KK, Moon-Grady AJ, Keller RL, Shiboski SC, Jelliffe-Pawlowski LL (2019) Impaired fetal environment and gestational age: what is driving mortality in neonates with critical congenital heart disease? J Am Heart Assoc 8(22):e013194 31726960 10.1161/JAHA.119.013194 PMC6915289

[17] Reddy RK, McVadon DH, Zyblewski SC, Rajab TK, Diego E, Southgate WM, Fogg KL, Costello JM (2022) Prematurity and congenital heart disease: a contemporary review. NeoReviews 23(7):e472–e485 35773510 10.1542/neo.23-7-e472

[18] Best KE, Tennant PWG, Rankin J (2017) Survival, by birth weight and gestational age, in individuals with congenital heart disease: a population-based study. J Am Heart Assoc 6(7):e005213 28733436 10.1161/JAHA.116.005213 PMC5586271

[19] Stoll BJ, Hansen NI, Bell EF, Walsh MC, Carlo WA, Shankaran S et al (2015) Trends in care practices, morbidity, and mortality of extremely preterm neonates, 1993–2012. JAMA 314(10):1039–1051 26348753 10.1001/jama.2015.10244 PMC4787615

[20] Desai J, Aggarwal S, Lipshultz S, Agarwal P, Yigazu P, Patel R, Seals S, Natarajan G (2017) Surgical interventions in infants born preterm with congenital heart defects: an analysis of the kids’ inpatient database. J Pediatr 191:103-109.e4 28964428 10.1016/j.jpeds.2017.07.015

[21] Morris M, Cleary JP, Soliman A (2015) Small baby unit improves quality and outcomes in extremely low birth weight infants. Pediatrics 136(4):e1007–e1015 26347427 10.1542/peds.2014-3918

[22] Fathi O, Nelin LD, Shepherd EG, Reber KM (2022) Development of a small baby unit to improve outcomes for the extremely premature infant. J Perinatol 42(2):157–164 33712714 10.1038/s41372-021-00984-0 PMC7952830

[23] Goldshtrom N, Vasquez AM, Chaves DV, Bateman DA, Kalfa D, Levasseur S, Torres AJ, Bacha E, Krishnamurthy G (2023) Outcomes after neonatal cardiac surgery: the impact of a dedicated neonatal cardiac program. J Thorac Cardiovasc Surg 165(6):2204-2211.e4 35927084 10.1016/j.jtcvs.2022.06.013

[24] Katz JA, Levy PT, Butler SC, Sadhwani A, Lakshminrusimha S, Morton SU, Newburger JW (2023) Preterm congenital heart disease and neurodevelopment: the importance of looking beyond the initial hospitalization. J Perinatol. 10.1038/s41372-023-01687-4 37179381 10.1038/s41372-023-01687-4

