# Supplementary material for: Double Jeopardy: A Distinct Mortality Pattern Among Preterm Infants with Congenital Heart Disease
Source: Pediatr Cardiol. 2024 Jun 12;46(4):939–46. doi: 10.1007/s00246-024-03519-4 (PMC11903637; doi:10.1007/s00246-024-03519-4)
Supplement: Supplementary file 1 — Supplementary file1 (DOCX 19 KB) [file 246_2024_3519_MOESM1_ESM.docx]

**Supplemental Table 1:** 1-Year survival of neonates with and without CCHD by gestational age

|  | **Proportion survived (95% CI)** | | **Risk difference** | |
| --- | --- | --- | --- | --- |
|  | **Neonates with CCHD**  **(n= 3,619)** | **Neonates without CCHD**  **(n=2,650,634)** | **Crude**  **(95% CI)** | **Adjusted***  **(95% CI)** |
| Gestational age |  |  |  |  |
| 21 weeks | 16.7 (3.5, 52.4) | 18.1 (17.3, 18.9) | 1.4 (-19.7, 22.5) | 1.2 (-17.9, 20.3) |
| 22 weeks | 12.5 (2.7, 41.7) | 25.5 (24.7, 26.3) | 13.0 (-3.2, 29.2) | 9.2 (-18.1, 36.5) |
| 23 weeks | 32.4 (19.1, 49.5) | 47.4 (46.6, 48.2) | 15.0 (-0.1, 30.1) | 16.2 (4.0, 32.0) |
| 24 weeks | 53.2 (40.6, 65.4) | 68.2 (67.6, 68.8) | 15.0 (2.6, 27.4) | 14.2 (13.7, 27.1) |
| 25 weeks | 51.6 (39.2, 63.7) | 79.3 (78.8, 79.8) | 27.8 (15.5, 40.0) | 23.5 (10.9, 36.2) |
| 26 weeks | 53.2 (42.9, 63.3) | 86.2 (85.8, 86.6) | 32.9 (22.7, 43.1) | 30.0 (19.5, 40.4) |
| 27 weeks | 53.1 (42.1, 63.8) | 90.6 (90.3, 90.9) | 37.5 (26.6, 48.4) | 32.2 (21.1, 43.4) |
| 28 weeks | 54.5 (45.5, 63.1) | 94.1 (93.8, 94.3) | 39.6 (30.8, 48.4) | 37.5 (28.4, 46.5) |
| 29 weeks | 57.5 (48.4, 66.1) | 95.6 (95.5, 95.8) | 38.1 (29.3, 47.0) | 35.6 (26.5, 44.6) |
| 30 weeks | 61.8 (54.1, 69.0) | 96.8 (96.7, 96.9) | 35.0 (27.6, 42.4) | 31.9 (24.4, 39.3) |
| 31 weeks | 54.4 (47.1, 61.5) | 97.5 (97.4, 97.6) | 43.1 (35.9, 50.3) | 37.9 (30.5, 45.3) |
| 32 weeks | 65.8 (59.6, 71.6) | 98.2 (98.1, 98.2) | 32.3 (26.4, 38.3) | 28.2 (22.4, 34.0) |
| 33 weeks | 61.9 (56.3, 67.4) | 98.7 (98.6, 98.7) | 36.7 (31.1, 42.3) | 31.7 (26.2, 37.2) |
| 34 weeks | 71.8 (67.7, 75.5) | 99.0 (99.0, 99.0) | 27.2 (23.3, 31.1) | 23.1 (19.4, 26.7) |
| 35 weeks | 73.1 (69.5, 76.4) | 99.2 (99.2, 99.2) | 26.2 (22.7, 29.6) | 20.9 (17.7, 24.0) |
| 36 weeks | 76.8 (74.0, 79.3) | 99.5 (99.5, 99.5) | 22.7 (20.1, 25.3) | 17.2 (15.0, 19.5) |

*adjusted for sex, multiple gestation and IUGR (z-score for BW< -1.3)

CCHD = cyanotic congenital heart disease, IUGR = intrauterine growth restriction

**Supplemental Table 2:** Early death of neonates with and without CCHD by gestational age

|  | **Proportion of early death (95% CI)^#^** | | **Risk difference** | |
| --- | --- | --- | --- | --- |
|  | **Neonates with CCHD**  **(n= 1,181)** | **Neonates without CCHD**  **(n= 71,605)** | **Crude**  **(95% CI)** | **Adjusted***  **(95% CI)** |
| Gestational age |  |  |  |  |
| 21 weeks | 100 | 95.0 (94.5, 95.5) | NA | NA |
| 22 weeks | 100 | 87.4 (86.7 (88.1) | NA | NA |
| 23 weeks | 68.0 (46.7, 83.7) | 65.7 (64.7, 66.7) | 2.3 (-16.1, 20.6) | 2.4 (-15.8, 20.6) |
| 24 weeks | 72.4 (52.8, 86.0) | 51.4 (50.2, 52.6) | 21.0 (4.7, 37.3) | 20.8 (4.3, 37.4) |
| 25 weeks | 61.3 (42.7, 77.1) | 46.4 (45.0, 47.7) | 14.9 (-2.3, 32.1) | 13.3 (-4.5, 31.0) |
| 26 weeks | 72.1 (56.5, 83.7) | 44.3 (42.8, 45.9) | 27.8 (14.3, 41.3) | 28.7 (15.1, 42.4) |
| 27 weeks | 60.5 (43.9, 75.0) | 45.3 (43.6, 47.1) | 15.2 (-0.4, 30.8) | 16.3 (0.5, 32.0) |
| 28 weeks | 57.1 (43.7, 69.6) | 44.8 (43.0, 46.7) | 12.3 (-0.7, 25.4) | 13.6 (0.4, 26.7) |
| 29 weeks | 60.8 (46.6, 73.4) | 43.8 (41.9, 45.8) | 17.0 (3.4, 30.5) | 18.3 (4.7, 31.8) |
| 30 weeks | 52.4 (39.9, 64.6) | 46.7 (44.7, 48.8) | 5.6 (-6.9, 18.1) | 6.9 (-5.6, 19.5) |
| 31 weeks | 63.9 (52.9, 73.6) | 45.3 (43.3, 47.3) | 18.5 (8.0, 29.1) | 18.8 (8.3, 29.4) |
| 32 weeks | 48.2 (37.5, 59.0) | 45.2 (43.3, 47.1) | 3.0 (-7.0, 13.9) | 2.8 (-8.2, 13.8) |
| 33 weeks | 49.5 (40.3, 58.9) | 41.8 (39.9, 43.7) | 7.8 (-1.7, 17.2) | 7.7 (-1.9, 17.3) |
| 34 weeks | 42.8 (34.9, 51.0) | 35.7 (34.1, 37.2) | 7.1 (-1.1, 15.3) | 7.8 (-0.5, 16.1) |
| 35 weeks | 33.7 (27.0, 41.2) | 33.1 (31.7, 34.6) | 0.6 (-6.6, 7.8) | 0.3 (-6.9, 7.5) |
| 36 weeks | 30.8 (25.1, 37.2) | 28.7 (27.5, 29.9) | 2.2 (-4.0, 8.3) | 1.0 (-5.0, 7.0) |

**^#^**The denominator is all deaths, i.e. proportion of early deaths relative to all deaths, early death was defined as death before day of life three

*adjusted for sex, multiple gestation and IUGR (z-score for BW< -1.3)

CCHD = cyanotic congenital heart disease, IUGR = intrauterine growth restriction

NA = not available
